# EP2-PKA signaling is suppressed by triptolide in lipopolysaccharide-induced microglia activation

**DOI:** 10.1186/s12974-015-0275-y

**Published:** 2015-03-14

**Authors:** Ting Zhang, Xiaoli Gong, Guanzheng Hu, Xiaomin Wang

**Affiliations:** Department of Neurobiology, Capital Medical University, Beijing Institute for Brain Disorders and Key Laboratory for Neurodegenerative Disorder, Ministry of Education, No. 10 Xitoutiao, Youanmenwai, Fengtai District Beijing, 100069 China; Department of Physiology, Capital Medical University, No. 10 Xitoutiao, Youanmenwai, Fengtai District Beijing, 100069 China

**Keywords:** Triptolide, EP2, Microglia, Neuroinflammation, Nitric oxide

## Abstract

**Background:**

Microglia are key players for the inflammatory responses in the central nervous system. Suppression of microglial activation and the resulting production of proinflammatory molecules are considered a promising strategy to alleviate the progression of neurodegenerative disorders. Triptolide was demonstrated as a potent anti-inflammatory compound both *in vitro* and *in vivo*. The present study explored potential signal pathways of triptolide in the lipopolysaccharide (LPS)-induced inflammatory response using primary rat microglial cells.

**Findings:**

Microglial cells were pretreated with triptolide and stimulated with LPS. To investigate the anti-inflammatory effect of triptolide, we used Griess reagent and Western blot for NO release and iNOS expression, respectively. Moreover, we applied microglia-conditioned medium to neuronal cells and used the MTS assay to test cell viability. We found that triptolide inhibited LPS-induced NO and iNOS synthesis in microglial cells, which in turn protected neurons. To evaluate the involvement of the EP2 pathway, we used real-time PCR and Western blot to determine EP2 expression. We found that LPS induced a large increase in EP2 expression in microglia, and triptolide almost completely inhibited LPS-induced EP2 expression. Using the selective EP2 agonist butaprost and the EP2 antagonist AH6809, we determined that triptolide inhibited LPS-stimulated NO production in microglia mainly through the EP2 pathway. Additionally, by further treating triptolide-treated microglia with the downstream PKA-specific activator 6-Bnz-cAMP or the Epac-specific activator 8-pCPT-2-O-Me-cAMP, we found that 6-Bnz-cAMP but not 8-pCPT-2-O-Me-cAMP increased NO production in triptolide-LPS treated microglia. These results indicate that the EP2-PKA pathway is very important for triptolide’s effects.

**Conclusions:**

Triptolide inhibits LPS-stimulated NO production in microglia via a signaling mechanism involving EP2 and PKA. This finding may help establish the pharmacological function of triptolide in neurodegenerative disorders. Moreover, the observation of inflammatory EP2 signaling in primary microglia provides important evidence that EP2 regulates innate immunity in the central nervous system.

**Electronic supplementary material:**

The online version of this article (doi:10.1186/s12974-015-0275-y) contains supplementary material, which is available to authorized users.

## Findings

Microglia, the immune-like cells of the brain, play an important role in inflammatory responses in the central nervous system (CNS) [1]. The modulation of microglial activation and their production of pro-inflammatory mediators and cytokines could also be a promising strategy to alleviate the progression of neurodegenerative disorders [2-5]. The cyclooxygenase-2 (COX-2) pathway mediates the main inflammatory responses in microglia and is thus a very attractive target for researchers and drug developers [6,7]. However, the deleterious cardiovascular and cerebrovascular side effect of sustained COX-2 inhibition has led to the investigation of its downstream targets [8]. Among these targets, prostaglandin E_2_ (PGE_2_) signaling via its EP2 receptor subtype appears to be a major mediator of inflammatory and anaphylactic reactions within both the periphery and brain [9]. EP2 upregulation by lipopolysaccharide (LPS) contributes to cerebral oxidative damage and secondary neurotoxicity, effects that are usually accompanied by the induction of NO synthase (NOS) and cyclooxygenase (COX) activities [10]. EP2 receptor not only serves as a downstream target of COX2 but also acts to influence COX2 and iNOS expression. It was reported that iNOS and COX2 induction was completely absent in EP2 KO microglia after LPS treatment, indicating that EP2 was necessary for the induction of iNOS and COX2 after LPS stimulation [10]. Moreover, LPS-activated microglia-mediated neurotoxicity was completely abolished in cultures lacking microglial EP2, indicating that microglial EP2 was critical to LPS-activated microglia-mediated neurotoxicity *in vitro* [10].

Triptolide is the major active component of *Tripterygium* extracts and possesses potent anti-inflammatory and immunosuppressive properties [11]. Our group demonstrated for the first time that triptolide possesses potent neuroprotective properties both *in vitro* and *in vivo* [12]. In an LPS-challenged inflammation model of Parkinson’s disease (PD), intraperitoneal injection with triptolide for 24 days significantly improved the behavior of PD rats, decreased DAergic neuron death, and increased DA levels in the striatum [13]. Further study in primary cultured rat microglia indicated that triptolide inhibits LPS-induced microglial activation and suppresses COX-2 expression and PGE2 release [14]. In the current study, we investigated the main pathway of triptolide in LPS-induced inflammatory responses in primary rat microglial cells. We found that triptolide suppressed LPS-induced nitric oxide (NO) production and inducible NO synthase (iNOS) synthesis in primary rat microglial cells, which in turn protects neuronal cells from microglia-conditioned medium-induced cell injury. Moreover, triptolide inhibits LPS-stimulated NO production in microglia via a signaling mechanism involving EP2 and PKA.

Triptolide was generously provided by Professor Peng-FeiTu (School of Pharmaceutical Sciences, Peking University, Beijing, China). This white crystalline drug has a melting point of 226°C to 240°C and, for this study, was 98% pure, as evaluated by reverse-phase high pressure liquid chromatography. The following materials were used for these studies: LPS (Sigma-Aldrich, St. Louis, MO, USA), a rabbit polyclonal antibody to iNOS (Abcam, Cambridge, UK), a rabbit polyclonal antibody to EP2 (Cayman, MI, USA), a mouse monoclonal antibody to CD11b (Millipore, Billerica, MA, USA), a mouse monoclonal antibody to GAPDH (Sigma-Aldrich), butaprost (Cayman, MI, USA), AH6809 (Cayman, MI, USA), 6-Bnz-cAMP (BIOLOG Life Science Institute, Bremen, Germany), 8-pCPT-2-O-Me-cAMP (Millipore, Billerica, MA, USA), KT5720 (Life Technologies, Carlsbad, CA, USA), FBS (Hyclone, Logan, UT, USA), streptomycin and penicillin (Life Technologies), 0.2-ml syringe filters, 96- and 24-well tissue culture plates, 100-mm diameter dishes (Corning, NY, USA), Dulbecco’s modified Eagle’s medium (DMEM), and DMEM/F-12 (Life Technologies).

The immortalized murine BV2 microglial cell line was purchased from Cell Resource Center, Institute of Basic Medical Sciences, Chinese Academy of Medical Sciences (Beijing, China). MN9D cells were kindly provided by Dr. Bastian Hengerer (Novartis Institute for BioMedical Research, Basel, Switzerland). The two cell lines were maintained in 5% CO_2_ at 37°C in DMEM/F-12 supplemented with 10 % fetal bovine serum (FBS), 1% streptomycin, and penicillin. The primary rat microglial cells were prepared as described previously [14]. Briefly, cerebral cortices of 0- to 1-day-old SD rats, devoid of meninges and blood vessels, were dissociated by mild mechanical trituration. The isolated cells were seeded in 75-cm^2^ culture flasks (1.5 brains in a flask) in DMEM/F-12 containing 10% FBS, 1% penicillin, and streptomycin. The cultures were maintained at 37°C in a humidified atmosphere of 5% CO_2_/95% air. Fourteen days later, the microglia were separated from astrocytes by shaking the flasks at 180 rpm for 2 h. The purity of the enriched microglia was >95%, as identified by CD11b (dilution, 1:800) immunocytochemical staining. All of the results are expressed as the mean ± standard deviation (SD) of at least three independent experiments performed in duplicate. One-way analysis of variance (ANOVA) and Newman-Keuls multiple comparison tests were used to compare the groups. The differences were considered to be significant at *P* < 0.05. The experimental procedures in this study were approved by the Committee on Animal Care and Usage (Capital Medical University), and efforts were engaged to minimize the number of animal usage and suffering.

**Figure 1:**
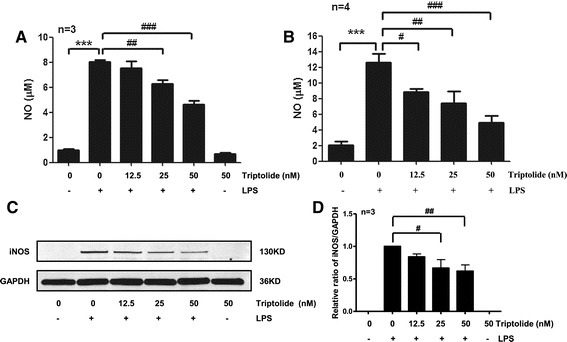
**Triptolide suppresses NO production and iNOS expression in LPS**-**activated microglia. (A)** Triptolide reduced NO production in BV2 cells. BV2 cells were stimulated with LPS (0.01 μg/mL) in the presence or absence of different concentrations of triptolide (0, 12.5, 25, 50 nM) for 24 h. At the end of the incubation period, the supernatants were collected for NO measurements. **(B) **Triptolide reduced NO production in primary rat microglial cells. Primary rat microglial cells were stimulated with LPS (0.01 μg/mL) in the presence or absence of triptolide (0, 12.5, 25, 50 nM) for 24 h. At the end of the incubation period, the supernatants were collected for NO measurements. **(C) **Triptolide reduced iNOS expression in primary rat microglial cells. Primary rat microglial cells were stimulated with LPS (0.01 μg/mL) in the presence or absence of triptolide (0, 12.5, 25, 50 nM) for 24 h. The cells were then lysed to detect iNOS expression by Western blot. **(D)** The protein levels were quantified relative to GAPDH levels and normalized to the LPS-stimulated group. ***P < 0.001. ^#^ *P* < 0.05, ^##^ *P* < 0.01, and ^###^ *P* < 0.001.

To confirm the anti-inflammatory effect of triptolide, we first investigated NO and iNOS production in the murine BV2 microglial cell line and in primary rat microglial cells. BV2 cells and primary rat microglial cells were cultured in 12-well plates and stimulated with LPS (0.01 μg/mL) for 24 h. At the end of the incubation period, supernatants were collected. NO measurements were made using the Griess Reagent System (Promega, Madison, WI, USA), and iNOS expression was measured by Western blot. In BV2 cells, LPS significantly induced NO release to 8 μM compared with 1 μM in naïve cells (Figure 1A). In primary microglia cells, LPS significantly induced NO release to 12 μM compared with 2 μM in naïve cells (Figure 1B). NO is synthesized by a family of NOS consisting of three isoforms: endothelial NOS (eNOS), neuronal NOS (nNOS), and inducible NOS (iNOS) [15]. Among them, NO produced from iNOS [16] promotes the development of neurodegenerative disorders associated with inflammation [17]. In the present study, the increase of NO correlated well with the induction of iNOS by LPS (Figure 1C, lane 2). BV2 cells and primary rat microglia both showed basal release of nitrite, which has been well demonstrated in previous reports by different laboratories [18-21]. Moreover, Ryu*et al*. reported that neither iNOS inhibitors nor nonselective nitric-oxide synthase inhibitors had any effect on the basal release of nitrite [22].

To investigate the inhibitory effect of triptolide on LPS-induced NO and iNOS synthesis, primary rat microglial cells were pretreated for 30 min with different concentrations of triptolide and subsequently stimulated with LPS. As shown in Figure 1A,B, triptolide dose dependently inhibited LPS-induced NO synthesis at concentrations from 12.5 to 50 nM. In BV2 cells, pretreatment with triptolide led to a dose-dependent inhibition on LPS-induced NO release by 6% (*P* < 0.05) at 12.5 nM, 22% (*P* < 0.01) at 25 nM, and 42% (*P* < 0.001) at 50 nM (Figure 1A). In primary microglial cells, pretreatment with triptolide led to a dose-dependent inhibition on LPS-induced NO release by 30% (*P* < 0.05) at 12.5 nM, 41% (*P* < 0.01) at 25 nM, and 61% (*P* < 0.001) at 50 nM (Figure 1B). As further illustrated in Figure 1C,D, the inhibitory effect of triptolide on NO synthesis was due to a dose-dependent inhibition of iNOS synthesis. iNOS synthesis was inhibited at a concentration of 25 nM and decreased significantly at a concentration of 50 nM triptolide. These results were consistent with a previous study in which Shen *et al*. reported that 30 nM triptolide inhibits NO production in LPS-activated macrophages [23].

**Figure 2:**
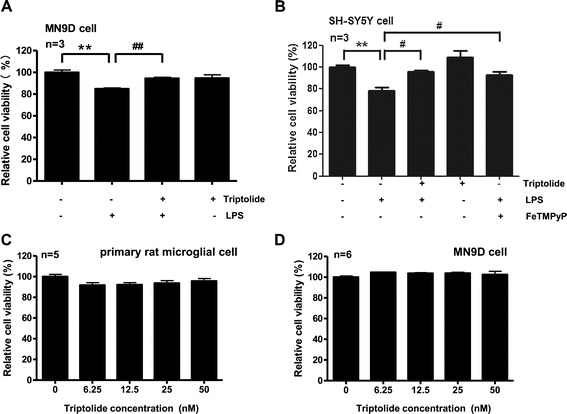
**Triptolide protects neuronal cells from the toxicity of LPS-stimulated microglia**-**conditioned medium. (A)** Triptolide attenuated the toxicity of conditioned medium from LPS-stimulated microglia to MN9D cells. MN9D cells treated with conditioned medium from LPS-unstimulated microglia, LPS-stimulated microglia with or without triptolide (50 nM), as well as triptolide alone (50 nM) treated microglia for 24 h. Cell viability was assessed by MTS assay. **(B) **Triptolide attenuated the toxicity of conditioned medium from LPS-stimulated microglia to SH-SY5Y cells. SH-SY5Y cells treated with conditioned medium from LPS-unstimulated microglia, LPS-stimulated microglia with or without triptolide (50 nM), triptolide alone (50 nM) treated microglia, as well as LPS-stimulated microglia with FeTMPyP (10 μM) for 72 h. Cell viability was assessed by MTS assay. **(C) **Triptolide did not change primary rat microglial cell viability. Primary rat microglial cells were treated with triptolide (0, 6.25, 12.5, 25, 50 nM) for 24 h. Cell viability was assessed by MTS assay. **(D) **Triptolide did not change MN9D cell viability. MN9D cells were treated with triptolide (0, 6.25, 12.5, 25, 50 nM) for 24 h. Cell viability was assessed by MTS assay. **P < 0.01. ^#^ *P* < 0.05 and ^##^ *P* < 0.01.

Activated microglia are capable of releasing neurotoxic molecules, such as proinflammatory cytokines and toxic oxygen and nitrogen species [24,25]. Accumulating evidence shows that activated microglia can damage or kill neurons *in vitro* by generating nitric oxide (NO) [26-29]. As microglia secreted increased amount of NO in response to LPS stimulation and triptolide inhibited this effect, we reasoned that triptolide may also reduce the neuronal toxicity of LPS-stimulated microglia-conditioned medium. MN9D cell, a fusion of neuroblastoma with mice embryonic ventral mesencephalic cell, was treated with the conditioned medium for 24 h, and an MTS assay was used to assess cell viability. The medium from microglia treated with only triptolide (50 nM) exhibited no toxicity. However, a 24 h exposure to medium from LPS-stimulated microglia caused a significant decrease of MN9D cell viability to 85% of that observed for MN9D cell exposed to medium from untreated microglia. In contrast, there was no significant change of MN9D cell viability between cells exposed to medium from microglia treated with triptolide (50 nM) followed by LPS and unstimulated microglia (Figure 2A). Since triptolide alone did not change the cell viability of MN9D cells or primary rat microglial cells (Figure 2C,D), the results indicated that triptolide-treated microglia attenuated the toxicity of the LPS-stimulated microglia-conditioned medium to MN9D cells.

We confirm the neuroprotective effect of triptolide in SH-SY5Y cells. The neuroblastoma cell line SH-SY5Y is often used in the cellular model of PD due to its dopaminergic ability [30,31]. As shown in Figure 2B, conditioned media from LPS-stimulated primary microglial cells significantly (*P* < 0.01) increased cell death of SH-SY5Y cells. Cell viability decreased to 76% of cells treated with medium from untreated microglia. This result is consistent with that reported by Liu *et al*. [32], Munch *et al*. [33], and Tseng *et al*. [34]. In contrast, the conditioned media from primary microglia cells pretreated with triptolide prior to LPS stimulation showed little neurotoxicity on SH-SY5Y cells, suggesting that triptolide suppresses microglia-mediated neurotoxicity.

To further investigate the role of NO release from microglia in toxicity of microglia to neuronal cells, we treated microglial cells with peroxynitrite (ONOO-) decomposition catalysts FeTMPyP [5,10,15,20-tetrakis(n-methyl-4′-pyridyl) porphinato iron (III) chloride]. NO reacts with superoxide (O2^−^), producing the highly reactive and toxic peroxynitrite (ONOO-) [35]. In the present study, we observed FeTMPyP at 10 μM blocked the LPS-induced microglial neurotoxicity in SH-SY5Y cells (Figure 2B), indicating peroxynitrite is a main mediator for the neurotoxicity of LPS-induced microglia.

The above findings that triptolide reduced the amount of proinflammatory factors in microglia and protected neurons from NO stimulation may help establish the pharmacological function of triptolide in neurodegenerative disorders.

**Figure 3:**
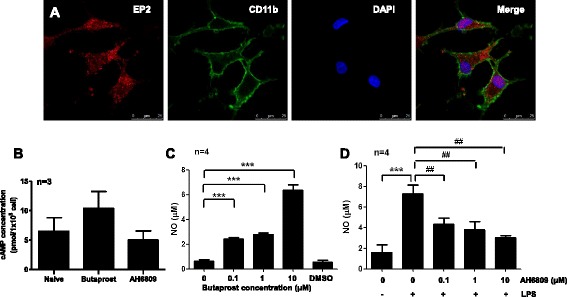
**EP2 signaling pathway regulates NO release in primary rat microglial cells. (A)** EP2 expression in primary rat microglial cells. Immunofluorescent staining of EP2 and CD11b, as well as the nuclear stain DAPI are shown (scale bar, 25 μm). **(B)** cAMP accumulation in rat microglia cells left untreated or treated with butaprost (10 μM) or AH6809 (10 μM) for 3 h (*n* = 3). **(C)** Butaprost stimulated NO production in resting microglia. Primary rat microglial cells were stimulated with different concentrations of butaprost (0, 0.1, 1, 10 μM) for 24 h. At the end of the incubation period, the supernatants were collected for NO measurements. **(D)** AH6809 suppressed NO production in LPS-activated microglia. Primary rat microglial cells were unstimulated or stimulated with LPS (0.01 μg/mL) in the presence or absence of AH6809 (0.1, 1, 10 μM) for 24 h. At the end of the incubation period, the supernatants were collected for NO measurements. ***P < 0.001 and ^##^ *P* < 0.01.

Microglial EP2 is critical to the neurotoxicity caused by the activation of cerebral innate immunity. To investigate whether EP2 is involved in the anti-inflammatory effect of triptolide in LPS-stimulated microglia, we first examined EP2 expression in primary rat microglial cells. We observed the basal expression of EP2 in resting microglia (Figure 3A). As a stimulatory G protein coupled receptor (GPCR), EP2 activation stimulates adenylate cyclase (AC). This effect results in an elevation of cytoplasmic cAMP levels, initiating multiple downstream events. To assess the functionality of the EP2 receptor in microglia, the cAMP level was determined by treating microglia with butaprost (10 μM), a selective EP2 agonist, or with AH6809 (10 μM), an EP2 antagonist. As shown in Figure 3B, there was a trend of increased intracellular cAMP accumulation in microglia treated with 10 μM butaprost for 3 h compared to control. By contrast, the treatment of microglia with 10 μM AH6809 for 3 h decreased the intracellular cAMP accumulation. Butaprost alone increased the basal level of microglia NO production (Figure 3C). This may be due to the iNOS expression influenced by butaprost; as Quan*et al*. reported, iNOS mRNA level in resting rat microglia was increased more than tenfold after 200 nM and 2 μM butaprost treatment [36]. Another EP2 agonist, ONO-AE1-259 was also reported to increase iNOS mRNA level in ileal tissue [37]. By contrast, AH6809 inhibited LPS-induced NO synthesis (Figure 3D). Since NO levels in the medium are also affected by proliferation and death of the treated microglia, we observed the cell viability after EP2 agonist and antagonist and the PKA agonist and antagonist treatment in BV2 cells and found no changes (Additional file 1: Figure S1). Thus, under an innate inflammatory stimulus, microglia EP2 signaling potentiated a classically activated proinflammatory response, inducing NO production.

**Figure 4:**
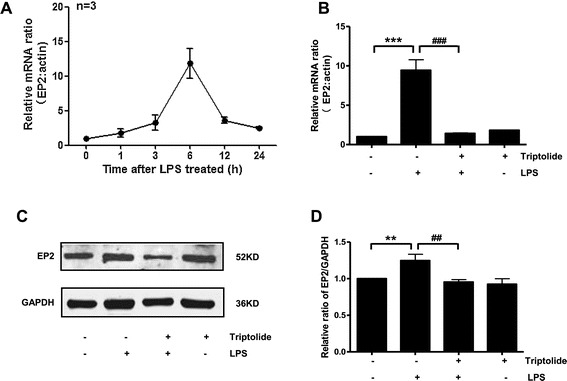
**Triptolide regulates EP2 expression in LPS**-**stimulated microglia. (A)** Rat primary microglia cells were stimulated with LPS (0.01 μg/mL) for 0, 1, 3, 6, 12, and 24 h. The cells were lysed for EP2 expression by real-time PCR analysis. **(B)** Rat primary microglia cells were stimulated with LPS (0.01 μg/mL) in the presence or absence of triptolide (50 nM) for 6 h. The cells were lysed for EP2 expression by real-time PCR analysis. **(C)** Primary rat microglial cells were stimulated with LPS (0.01 μg/mL) in the presence or absence of triptolide (50 nM) for 24 h. The cells were lysed for EP2 expression by Western blot. **(D)** The protein levels were quantified relative to GAPDH levels and normalized to the naïve group (untreated cells). **P < 0.01. ***P < 0.001. ^##^ *P* < 0.01 and ^###^ *P* < 0.001.

To evaluate the involvement of the EP2 pathway in triptolide’s effect, real-time PCR of EP2 was performed on a Stratagene Mx3000P (Agilent, Santa Clara, CA, USA) using the following parameters: 95°C for 10 min, followed by 40 cycles at 95°C for 15 s, 60°C for 1 min, and 72°C for 1 min using the specific primer sets. The following primer pairs were employed: 5′ - TTGCTCTTCTGTTCTCTGCCG - 3′ (upper, 538 to 558) and 5′ - CAGCTGAAGGTATGCGGTCC - 3′ (lower, 642 to 623) for the amplification of rat EP2 receptor (GenBank accession No. NM_031088) and 5′ - ATCGCTGACAGGATGCAGAAG - 3′ (upper, 925 to 945) and 5′ - AGAGCCACCAATCCACACAGA - 3′ (lower, 1,032 to 1,012) for the amplification of rat β-actin. Firstly, we observed the EP2 mRNA level at 1, 3, 6, 12, and 24 h after LPS induction and found that LPS augmented the EP2 expression significantly at 6 h (Figure 4A). The relative expression level compared with that of resting microglia increased to nearly 12-fold with LPS, which is similar as that reported by Noda *et al*. [38]. Moreover, we observed a prompt downregulation of EP2 mRNA level after 12-h LPS treatment. Johansson *et al*. reported that stimulation of macrophages with LPS (10 ng/ml) induced a rapid increase in EP2 mRNA within 1 h followed by a prompt downregulation by 6 h [39]. The different time of EP2 mRNA upregulation between macrophages and microglia maybe caused by their local microenvironment, as well as the differences between periphery and central inflammation, but the similar changes of EP2 expression induced by LPS indicate that EP2 expression is tightly regulated by inflammatory stimuli.

To investigate the effect of triptolide on LPS-regulated EP2 expression, microglia were pretreated for 30 min with triptolide (50 nM) and subsequently stimulated with LPS. As shown in Figure 4B, 50 nM triptolide almost completely inhibited LPS-induced EP2 expression. To confirm the triptolide inhibition of EP2 expression, we next monitored the protein level changes of EP2 by Western blot (Figure 4C,D). Although the changes in EP2 protein levels were not as dramatic as were observed for mRNA levels, triptolide did inhibit EP2 protein expression.

**Figure 5:**
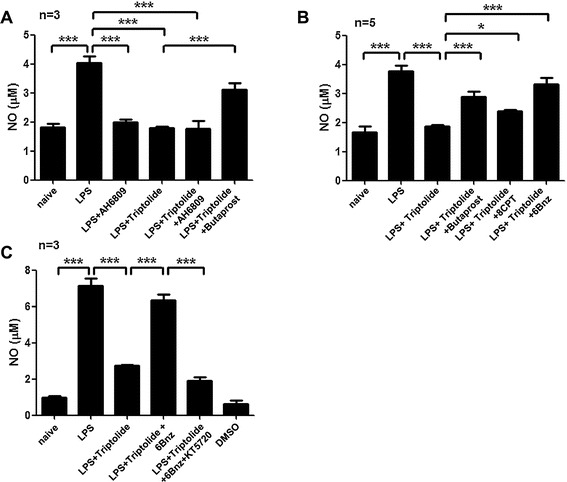
**EP2**-**PKA pathway is important in LPS**-**stimulated NO production and triptolide inhibition. (A)** Primary rat microglial cells were treated with LPS (0.01 μg/mL), LPS plus AH6809 (10 μM), LPS plus triptolide (50 nM), LPS plus triptolide plus AH6809, and LPS plus triptolide plus butaprost (0.1 μM) for 24 h. At the end of the incubation period, the supernatants were collected for NO measurements. **(B)** Primary rat microglial cells were treated with LPS (0.01 μg/mL), LPS plus triptolide (50 nM), LPS plus triptolide plus butaprost (0.1 μM), LPS plus triptolide plus 8-CPT (10 μM), or LPS plus triptolide plus 6Bnz (10 μM) for 24 h. At the end of the incubation period, the supernatants were collected for NO measurements. **(C)** BV2 cells were treated with LPS (0.01 μg/mL), LPS plus triptolide (50 nM), LPS plus triptolide plus 6Bnz (10 μM), and LPS plus triptolide plus 6Bnz (10 μM) plus KT5720 (1 μM) for 24 h. At the end of the incubation period, the supernatants were collected for NO measurements. *P < 0.05 and ***P < 0.001.

To confirm the necessary role of EP2, we treated primary rat microglial cells with butaprost/AH6809 and triptolide before LPS stimulation. As shown in Figure 5A, treatment with triptolide or AH6809 (10 μM) alone both decreased NO production in LPS-treated microglia to similar levels as were observed in normal microglia. Moreover, the combination of triptolide and AH6809 failed to further decrease NO production. Conversely, the addition of butaprost (0.1 μM) partially reversed the triptolide-induced inhibition of NO production (Figure 5A). Taken together, these results demonstrate that triptolide inhibits LPS-stimulated NO production in microglia via a signaling mechanism involving EP2. These results also indicate that triptolide regulation of the EP2 pathway is a potential therapeutic mechanism for the treatment of inflammation-related neurological disorders.

EP2 activation leads to increased levels of cytoplasmic cAMP, which then initiates multiple downstream events via the PKA or Epac pathways. To differentiate between these two pathways, we added the PKA-specific activator 6-Bnz-cAMP (6Bnz) or the Epac-specific activator 8-pCPT-2-O-Me-cAMP (8-CPT) to triptolide-treated microglia. We found that 6Bnz (10 μM) but not 8-CPT (10 μM) increased NO production in triptolide-LPS treated microglia (Figure 5B), indicating that EP2-PKA was mainly involved in the triptolide inhibition. This specificity was most likely due to the nature of LPS stimulation. Peters-Golden *et al*. reported that in rat alveolar macrophages, the down-modulation of LPS-induced TNF-α by PGE_2_ is dependent on cAMP-PKA activation. This was concluded given that the selective PKA activating cAMP analog 6Bnz but not the Epac-1 activating analog 8-CPT inhibited TNF-α production [40]. Their recent work also indicates that EP2-PKA, rather than Epac-1, is involved in the enhancement of LPS-induced NO [41]. This result suggests that the EP2-PKA pathway is very important for the anti-inflammatory effect of triptolide in microglia. More studies are needed to clarify whether the EP2-PKA signaling pathway is the dominant mediator of harmful EP2-mediated effects in microglia.

We further observe this effect in BV2 cells, and found 6Bnz (10 μM) increased NO production in triptolide-LPS treated microglia (Figure 5C). To confirm that PKA is indeed responsible for the inhibition of NO generation by triptolide, BV2 cells were pre-treated with the PKA inhibitor KT5720, and the PKA-specific activator 6Bnz was added before triptolide treatment. We found that 6Bnz (10 μM) increased NO production in triptolide-LPS treated microglia, KT5720 attenuated NO production to an extent similar to that observed with triptolide treatment (Figure 5C), suggesting that PKA is responsible for the triptolide inhibition.

The direct target of triptolide in the regulation of EP2-PKA pathway remains unknown. Liu *et al*. report that triptolide directly binds to human XPB, a subunit of the transcription factor TFIIH, leading to the inhibition of RNA polymerase II-mediated transcription in tumor cells [42]. However, these authors used higher concentrations of triptolide (1 to 100 μM) to inhibit tumor cell proliferation. We used lower concentrations of triptolide (no more than 50 nM) to suppress inflammation and to protect neurons in the brain. Therefore, different targets may exist for triptolide with respect to the modulation of the inflammatory response in the CNS. Recently, Shen *et al*. reported that triptolide inhibited TAK1 kinase activity by interfering with the formation of the TAK1-TAB1 complex, and that the binding affinity of triptolide to TAB1 was highly correlated with the inhibitory activity of triptolide against the MAPK pathway activation in macrophages [23]. Although macrophages and microglia are both tissue-resident immune cells during inflammation and the effective triptolide concentration was similar in both cell types, it remains to be investigated whether TAB1 is the target of triptolide in microglia.

## Conclusions

The present study demonstrates that triptolide inhibits LPS-stimulated NO production in microglia via a signaling mechanism involving EP2 and PKA. The finding that triptolide reduces the proinflammatory factors in microglia and protects neurons from inflammatory stimulation may help establish the pharmacological function of triptolide in neurodegenerative disorders. Moreover, the observation of inflammatory EP2 signaling in primary microglia also provides important evidence with respect to EP2 in the regulation of innate immunity in the CNS.

## Additional file

Additional file 1: Figure S1. Microglia proliferations did not change after pharmacological treatments. BV2 cells treated with butaprost (0.1 μM), AH6809 (10 μM), 8-CPT (10 μM), 6Bnz (10 μM), KT5720 (1 μM), and DMSO (0.1%) for 24 h. Cell viability was assessed by MTS assay.

## Abbreviations

LPS: lipopolysaccharide
NO: nitric oxide
iNOS: inducible NO synthase
COX-2: cyclooxygenase-2
PGE_2_: prostaglandin E_2_
CNS: central nervous system
PD: Parkinson’s disease
GPCR: G protein coupled receptor
AC: adenylate cyclase
PKA: protein kinase A
Epac: exchange proteins that are directly activated by cAMP
6Bnz: 6-Bnz-cAMP
8-CPT: 8-pCPT-2-O-Me-cAMP
MAPK: mitogen-activated protein kinase
DMEM: Dulbecco’s modified Eagle’s medium
FBS: fetal bovine serum
GAPDH: glyceraldehyde-3-phosphate dehydrogenase
PBS: phosphate-buffered saline
PCR: polymerase chain reaction
SD: standard deviation
