# Supplementary figures and images for: EP2-PKA signaling is suppressed by triptolide in lipopolysaccharide-induced microglia activation
Source: J Neuroinflammation. 2015 Mar 14;12:50. doi: 10.1186/s12974-015-0275-y (PMC4364339; doi:10.1186/s12974-015-0275-y)

## Slide 1
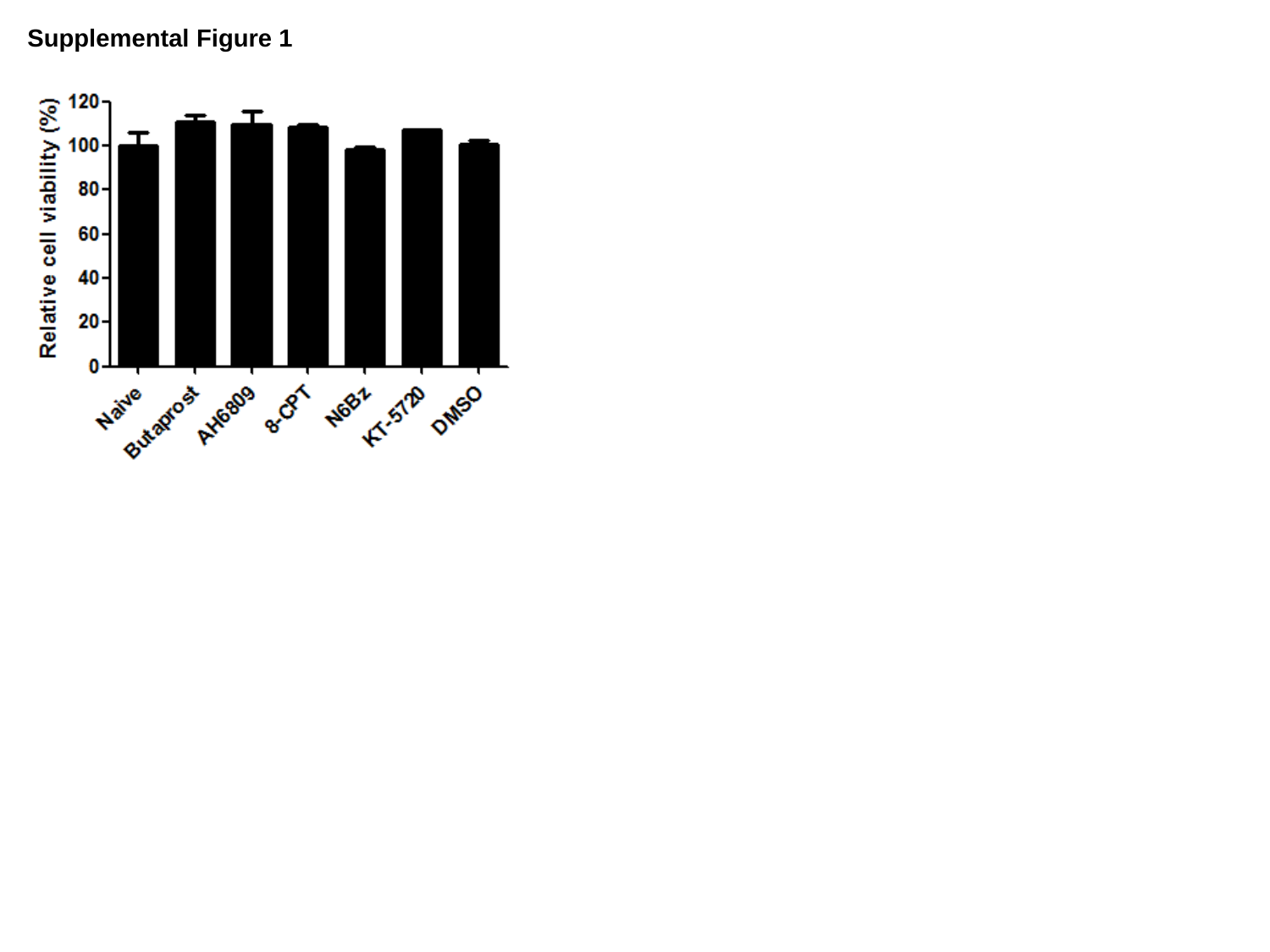

Supplemental Figure 1

Supplement: Additional file 1: Figure S1. — Microglia proliferations did not change after pharmacological treatments. BV2 cells treated with butaprost (0.1 μM), AH6809 (10 μM), 8-CPT (10 μM), 6Bnz (10 μM), KT5720 (1 μM), and DMSO (0.1%) for 24 h. Cell viability was assessed by MTS assay. [file 12974_2015_275_MOESM1_ESM.ppt]
